# ‘We All Try to Imagine What It's Like for Someone to Be Dependent’: Attitudes Towards Drug Users Among Medical Students in the United Kingdom and Its Impact on Their Professional Identity Formation

**DOI:** 10.1111/tct.70473

**Published:** 2026-07-07

**Authors:** Holly Melvin, Dilmini Karunaratne, Jason W. Boland

**Affiliations:** ^1^ Manchester University Foundation Trust Manchester UK; ^2^ Centre for Medical Education University of Dundee Dundee UK; ^3^ Palliative Medicine and Education, Wolfson Palliative Care Research Centre, Hull York Medical School University of Hull Hull UK

**Keywords:** attitudes, drug dependency, drug users, illicit drugs, methadone, professional identity formation, stigma

## Abstract

**Background:**

Evidence suggests that some doctors and medical students hold stigmatising attitudes towards drug users. This area remains underexplored in the United Kingdom (UK), and current medical students' attitudes to drug users are unknown.

**Aims:**

This study aimed to explore the UK medical students' attitudes towards drug use, factors influencing these attitudes and their impact on professional identity formation.

**Methods:**

A mixed methods study used a questionnaire and interviews in two UK medical schools.

**Results:**

Data were collected from 62 questionnaires and 12 interviews. Most UK medical students (71.0%, *n* = 44) viewed addiction as a chronic health condition and supported providing optimal care (91.8%, *n* = 56). However, support for funding addiction services declined during training (100%–60%), reflecting growing stigma. Prolonged heroin (90.2%, *n* = 55) and cocaine use (88.6%, *n* = 54) were widely viewed as unacceptable, while responses regarding party drugs and methadone were mixed. Students' attitudes towards drug users were shaped by media, upbringing, peers, direct interactions with drug users and doctors' role modelling. These influences generally supported professional identity formation, although challenges remained. Related university teaching was widely perceived as insufficient, with reliance on hidden and informal curricula.

**Conclusion:**

While most medical students hold positive attitudes towards drug users, stigmatising views emerge during training, with declining support for treatment funding and worsening attitudes with seniority. Students' professional identity formation is shaped more by clinical encounters and role models than formal teaching. Current overreliance on hidden curricula creates inequitable learning experiences. These findings call for structured, interprofessional team‐based training to ensure compassionate, evidence‐based care for this vulnerable population.

## 1 Introduction

Drug use and dependence are an increasing health problem, with 310,863 adult patients in 2023–2024 accessing drug and alcohol treatment services in England [1]. Most of these services were sought for drug‐related issues, with only 30% for alcohol [1]. Given these high and increasing rates [1], the UK medical students will encounter many such patients as future healthcare practitioners.

Internationally, literature on drug use reveals that healthcare students hold mixed views [2]. Most (63.2%) believe that drug users are incapable of looking after themselves, a stigmatising attitude [2]. A large majority (91.0%) also believe that drug dependence is treatable [3] and rejects the idea that entering an addiction facility is a failure (90.8%) [2]. Amidst limited literature, a 2012 study reported that the UK students held stigmatising attitudes towards intravenous drug users [4]. Additionally, evidence suggests that doctors hold stigmatising attitudes that worsen with seniority [5]. These negative attitudes may impact medical students' attitudes towards drug users and their professional identity formation (PIF) as future doctors [6]. Research in this area remains limited within the UK context. Therefore, it is critical that attitudes of medical students and the factors impacting their PIF towards drug users are understood to better prepare them for providing high‐quality care to this patient group.

PIF describes an individual's transition to becoming a doctor and their interpretation of a good clinician [7]. PIF is a complex, evolving process, influenced by many factors, including clinical experiences, nonclinical experiences, values, morals, academic, sociocultural, religious factors and gender‐based roles [7]. PIF of medical students takes place largely through socialisation [8]. Socialisation is when a person learns to function within a particular group through internalisation of values and norms [8]. Socialisation and PIF towards drug users take place in clinical and nonclinical environments, impacted by positive and negative role models [9]. Substance misuse‐related learning objectives are implemented in all UK medical undergraduate curricula [10]. However, interviews and curricula mapping underscored the need for more focus on behaviour and attitudes than theory, which may be more influential on PIF [10].

Students' attitudes towards drug use must be understood within the current global context, where drug users widely express concerns about receiving care that does not meet their expectations [11, 12].

The sociocultural learning theory [13], which emphasises learning as a social process shaped by interactions with others and influenced by cultural context, offers a useful lens to explore the factors shaping medical students' perceptions of drug users. It also helps explain how students collaboratively co‐construct knowledge and internalise new understandings, contributing to their evolving professional identity as future doctors. Informed by this theoretical perspective, the present study was undertaken to examine the UK medical students' attitudes towards drug use, factors shaping these attitudes and how these influence their PIF.

Research objectives are as follows:

i) To explore attitudes of the UK medical students towards drug use.
ii) To investigate the factors that influence these attitudes.
iii) To examine how these attitudes inform their PIF in relation to drug use.
iv) To explore student perceptions on current medical school teaching on illicit drug use and identify perceived educational needs in this area.

## 2 Materials and Methods

### 2.1 Methodology

A mixed methods research approach was used to integrate both qualitative and quantitative perspectives on this topic [14]. The quantitative element helped identify areas for deeper exploration during the qualitative interviews. The qualitative aspect of the study was guided by an interpretivist stance, recognising reality as subjective and allowing for diverse interpretations to construct meaning within the researched community [15].

### 2.2 Ethics

Hull York Medical School (HYMS) Ethics Committee approval obtained on 29 November 2021 (REC 21/22 13).

### 2.3 Study Population

The study sampled medical students at two UK medical schools, the Hull York Medical School and the University of Manchester.

### 2.4 Data Collection Methods

The study employed an online questionnaire followed by semistructured in‐depth interviews as the primary data collection methods. Invitation for the online questionnaire was advertised centrally through a newsletter, notice boards and during teaching sessions. Interested students were given further information and access to the questionnaire link, where they initially consented to participation. The online questionnaire was a validated tool, ‘Public attitudes towards people with drug dependence and people in recovery questionnaire’, developed by the UK Drug Policy Commission [16]. It explored individual attitudes towards substance users, perceived acceptability of different drugs and personal experiences, which were relevant to medical students. Additionally, a pilot test with five medical students ensured that the online questionnaire contained no errors, was understood well and was user‐friendly.

This was followed by individual semistructured interviews, chosen for their ability to explore participants' opinions in depth [17], while offering greater privacy than focus groups. The interview guide (Appendix S1) was developed by adopting relevant open‐ended questions tailored to the research aim.

### 2.5 Data Analysis

#### 2.5.1 Analysis of Questionnaire Data

The questionnaire data were analysed using Qualtrics software. Questions were individually analysed to statistically summarise participant responses and to find significant relationships. The *p*‐values less than 0.05 were interpreted as statistically significant.

#### 2.5.2 Analysis of Interview Data

Interviews were transcribed verbatim. Braun and Clarke's [18] inductive approach to thematic analysis was followed. It consisted of six steps: familiarise with data, generate initial codes, search for themes, review themes, define and name themes and produce report. Reflexivity was key, and the researcher kept a reflective journal to document personal views and how they evolved throughout the study. This approach ensured that interpretations were grounded in the data collected. Credibility was maintained through employing a transparent approach to coding and its systematic application to all transcripts [19].

## 3 Results

### 3.1 Questionnaire Findings

Sixty‐two students completed the questionnaire. The majority of participants (71%, *n* = 44 participants) attended the University of Manchester, while the remaining 29.0% (*n* = 18 participants) attended the Hull York Medical School. A total of 38.7% (*n* = 24 participants) were identified as male, 58.1% (*n* = 36 participants) female and 3.2% (*n* = 2 participants) nonbinary. The ethnicity of respondents was white (54.8%), Asian (29.0%), mixed (11.3%) and black (3.2%) with 1.6% choosing not to disclose. In terms of age distribution, 11.2% were between 18–20 years, 72.6% 21–23 years and 16.2% > 24 years. The distribution of study participants according to year groups one to five was 6.5% (*n* = 4, Year 1), 8.1% (*n* = 5, Year 2), 22.6% (*n* = 14, Year 3), 37.1% (*n* = 23, Year 4) and 25.8% (*n* = 15, Year 5). There was no statistically significant difference in responses between medical schools. The key findings of the questionnaire are categorised into several main themes, as outlined below.

### 3.2 Students' Attitudes Towards Individuals With Drug Dependence

Most participants (91.9%, *n* = 57) strongly disagreed that drug users are bad people and disagreed (95.1%, *n* = 58) that drug users do not deserve sympathy. A majority (78.7%, *n* = 48) strongly agreed that people with a history of drug dependence are demonised in the media. However, the agreement declined with the academic year, from 100% in Year‐1 to 73.3% in Year‐5. The statistically significant relationship (*p* = 0.03) between year of study and decreasing agreement was meaningful in practice (Cramér's V effect size 0.3).

### 3.3 Students' Perceptions on Treating Patients With Drug Dependence

The majority of participants (71.0%, *n* = 44) strongly agreed that drug dependence is like any other chronic health problem. Additionally, a majority (91.8%, *n* = 56) also agreed that healthcare professionals have a responsibility to provide the best possible care for people who use drugs. Similarly, most participants (70.5%, *n* = 43) strongly disagreed that increasing spending on substance use services is a waste of money. The responses to this question showed a statistically significant association with both gender (*p* < 0.00001 and Cramér's V effect size 0.6) and year group (*p* = 0.0375 and Cramér's V effect size 0.365). Female respondents were more likely to disagree with the statement (85.7%) compared with males (50%). The agreement that increasing spending is worthwhile declined across training, from 100% in first‐year students to 60% among final‐year students.

### 3.4 Students' Perceptions on the Nature of Drug Dependence

Most participants (91.9%, *n* = 57) agreed that virtually anyone can become dependent on drugs. A minority (26.2%, *n* = 16) agreed that people can never completely recover from drug dependence, and it is easy to distinguish drug users from others (24.2%, *n* = 15), while the rest disagreed.

### 3.5 Student's Perceived Acceptability of Different Drug Types and Usage

On a scale of 1–10 (1 = *very acceptable*, 10 = *not at all acceptable*), the majority of respondents, 88.6% (*n* = 54) and 90.2% (*n* = 55), considered daily cocaine use and daily long‐term heroin use (10 + years) as highly unacceptable respectively by scoring 8–10 on the scale. Perceptions of methadone use were mixed, with 49.3% (*n* = 30) rating it as acceptable (scores 1–3), reflecting divided views on whether methadone users are ‘recovered’ (36.1%, *n* = 22 agreed; 39.3%, *n* = 24 disagreed). Opinions on cannabis, ecstasy and other illegal stimulants also varied widely, indicating no clear consensus among students (Table 1).

**TABLE 1:** Results of perceived acceptability of drugs (% and number of responses).

	Smoking cannabis a few times a week	Using cocaine every day	Using methadone for 10 years or more	Using heroin on a daily basis for 10 years or more	Using ‘party drugs’ (e.g., ecstasy/other illegal stimulants) at the weekend
1 = *very acceptable*	14.8% (9)	1.6% (1)	19.7% (12)	1.6% (1)	9.8% (6)
2	9.8% (6)	—	14.8% (9)	—	6.6% (4)
3	11.5% (7)	—	14.8% (9)	1.6% (1)	11.5% (7)
4	9.8% (6)	1.6% (1)	19.7% (12)	—	11.5% (7)
5	13.1% (8)	—	11.5% (7)	—	8.2% (5)
6	9.8% (6)	4.9% (3)	1.6% (1)	1.6% (1)	9.8% (6)
7	9.8% (6)	3.3% (2)	6.6% (4)	3.3% (2)	11.5% (7)
8	11.5% (7)	6.6% (4)	3.3% (2)	3.3% (2)	11.5% (7)
9		6.6% (4)	1.6% (1)	6.6% (4)	8.2% (5)
10 = *not at all acceptable*	9.8% (6)	75.4% (46)	1.6% (1)	80.3% (49)	11.5% (7)
11 = *do not know*			4.9% (3)	1.6% (1)	—

*Note:* ‘—’, no responses for the section.

### 3.6 Findings of Interviews

Twelve interviews were conducted. Four themes were generated through thematic analysis. These are described (Appendix S2) and presented as an interpretive narrative below, supported by participant quotations. Quotations are followed by the pseudonyms students chose for themselves.

#### 3.6.1 Theme 1: Factors Shaping Perceptions and Attitudes Towards Patients Who Use or Are Dependent on Illicit Drugs

Participants' attitudes towards substance use were shaped by direct interactions with patients, fostering empathy and understanding towards them. Exposure to diverse opinions of peers, friends and partners who use illicit drugs also influenced their views and positively shaped their PIF as future doctors.

The medical school played a key role in shifting initial negative perceptions, with positive role modelling by peers and doctors. While some unsatisfactory doctor–patient interactions occurred with substance users, they did not significantly impact participants' attitudes. The influence of allied healthcare professionals was minimal, and university teaching had mixed effects. However, working in a healthcare role outside their studies positively shaped their attitudes towards substance use (Table 2).

**TABLE 2:** Quotes illuminating the factors influencing participant attitudes towards illicit drug users.

“I've almost lost a few friends … to illicit drugs … I have a lot more compassion and empathy” Lance
“I also have a family member who is addicted to illicit drugs … that's had a significant effect on me … As a child, … it was ingrained in me that it's … a choice … When that's not the case” Saskia
“All doctors I've spoken to have never spoken down on patients that use illicit drugs. They'll emphasise that they are in a position of vulnerability, so it's important to have extra care for them … it's made me aware you have to be very open‐minded” Julien
“It's definitely more senior doctors who will … be very dismissive, they'll just want to get them out … comments of he's a druggie … it can definitely influence the culture around how staff view the dependency … if a consultant making jokes … it sets the tone” Elif
“One of the lecturers … was really clear on it not necessarily being a fault, and the addiction being a condition that needed help, which has really influenced my opinion … I'll be much less judgemental … I think it's definitely developed my empathy”Sue

#### 3.6.2 Theme 2: Students' Understanding of the Nature of Addiction and Illicit Drug Use

Many students attributed the causes of drug use to social circumstances and mental health issues. They frequently compared drug use to other health conditions, often reflecting on their own experiences and those of peers, family and other patients. The students discussed societal stigma and attitudes towards drug users, with all agreeing that individuals with drug use deserve care. While some believed that this care should be equivalent to that provided to nonillicit drug users, others felt that those with drug use required additional support (Table 3).

**TABLE 3:** Quotes illuminating the students' understanding of the nature of addiction and illicit drug use.

“Sometimes (they) do not have a choice when they get into these habits, it's complicated … (they) had depression, had anxiety, had difficult social situations, and their way of coping with it was illicit drug use” Elif
“I had been on a gastroenterology ward, where there's a lot of alcohol addiction … I think you realise how hard and how much of a struggle it is” Saskia
“I think society has this overly criminal view of drug usage. I think a combination of individual pre‐conceived notions and prejudices … is a barrier to effective healthcare” David
“I do not think that they are undeserving at all … addiction is an illness, and it needs support and help” Saskia

#### 3.6.3 Theme 3: Perceived Gaps and Opportunities in Medical School Teaching on Illicit Drug Use

Some students expressed satisfaction with the teaching on drug use and dependence, finding the quantity and content suitable. However, many others were dissatisfied, citing issues with both the quantity, content and the nature of the teaching sessions. Students also highlighted learning from the ‘hidden and informal curriculum’. They proposed various changes for university teaching on illicit drug use, with a common suggestion of having timetabled exposure to interact with substance users (Table 4). They emphasised that exposure to patients who take illicit drugs enables students to appreciate the significance of the issue and helps accelerate their learning. As one participant explained, ‘As much as we all try to imagine what it's like for someone to be dependent, you'd never really know unless you speak to somebody’ (Josephine).

**TABLE 4:** Quotes illuminating perceived gaps and opportunities in medical school teaching on illicit drug use.

“I think it's fine (undergraduate teaching on this area). We had a case on schizophrenia, and there was a lot of talk about illicit drug use … from quite early on, we have done a little bit on it” Annabelle
“I do not think there was any sort of teaching at all … about illicit drug use and trying to destigmatise that … We never had any communication sessions … it's kind of appalling … there needs to be more teaching” Elif
“I think a lot of the information we are expected to find out ourselves in our placements … a little more guidance might mean that we'll know what to ask when we meet the patient that has got an illicit drug dependence” Jason

*As much as we all try to imagine what it's like for someone to be dependent, you'd never really know unless you speak to somebody. Josephine*

#### 3.6.4 Theme 4: Student Aspirations for Caring for People Who Use Illicit Drugs

Although students observed occasional unsatisfactory treatment of drug users by senior medical staff, which contributed to a generally negative learning environment, most students described their vision of a ‘good doctor’ as someone who treats drug users empathetically, emphasising equal treatment, nonjudgmental care, compassion and attention to social factors. They also reflected on how they aspire to apply these principles in their future practice when treating substance users.

Try to be compassionate. Try to understand why they are using illicit drugs … if the patient does not feel listened to, they are a lot less likely to take on any help that you suggest. Sara

The doctors on the wards … have always been … really sensitive and empathetic. I'd like to be able to do what they do. Saskia

## 4 Discussion

**FIGURE 1:**
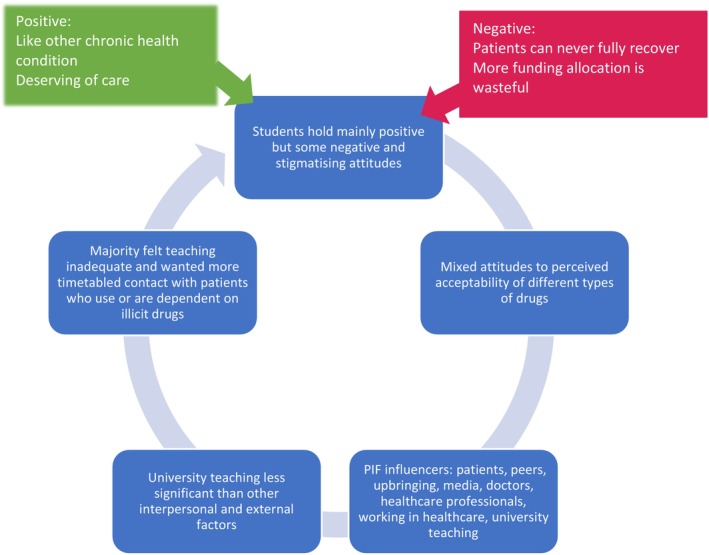
Factors influencing student attitudes towards illicit drug use and their professional identity formation concerning individuals who use drugs.

In light of the growing global and the UK prevalence of drug use [1] and the anticipated rise in patients with drug use related issues entering the healthcare system, this study was conducted among medical undergraduates to examine their attitudes, perceptions and the factors influencing their PIF in this domain. The study uncovered several practically relevant findings summarised in Figure 1 below.

### 4.1 Students' Opinions on and Attitudes to Patients Who Use Illicit Drugs and Drug Dependence

Both questionnaire and interview data revealed that many students hold positive nonstigmatising attitudes towards drug use. Nearly all questionnaire participants strongly disagreed that individuals dependent on drugs are inherently bad people, and interviews reflected similar nonjudgemental views. There was a clear trend that medical students often perceive drug dependence as similar to other chronic health conditions aligning with international research [2]. These perceptions were shaped by a range of influences, including family, peers, partners, medical teachers and academic discussions on substance use during training. Most notably, students emphasised the value of firsthand experiences, whether through peers or close family members or direct interactions with individuals who use drugs during clinical placements in influencing their views.

The questionnaire, however, revealed some stigmatising attitudes not captured in the interviews. Notably, over a quarter of respondents believed that complete recovery from drug dependence is unattainable. Towards the senior years of training, attitudes towards drug users became more stigmatising, with decreasing agreement on media demonising drug users and increasing views that more allocation of funds for treatment in this area is wasteful. Interviews revealed that the learning culture was also shaped by the attitudes of clinical staff, especially the senior medical staff. Although negative attitudes towards people who use drugs were not uncommon in the clinical environment, these were often viewed critically by students. These contrasting experiences highlight the significant sociocultural influences on medical students' learning and PIF towards caring for drug users [13]. This mixed perspective correlated with international research [2, 3] and highlights the ongoing need for medical educators to address and improve students' attitudes and PIF regarding drug use.

### 4.2 Perceived Acceptability of Illicit Drug Use

The mixed response regarding the perceived acceptability of methadone is interesting as this can influence how future healthcare professionals approach the treatment of substance users. Disparities in perceived acceptability of different illicit drugs were also uncovered. While not all students found cannabis or ‘party drugs’ acceptable, many more students found these acceptable compared to cocaine and heroin. This correlates with findings from a systematic review that overall prevalence of lifetime cannabis use in medical students is 31.4% [20], meaning it is highly likely that some of the students answering will have used cannabis, or know people who do, potentially increasing its perceived acceptability.

### 4.3 Students' Opinions on Current Teaching on Illicit Drug Use and Dependence

Students had strong opinions on current medical school curricula. The impact of medical school education has on students' PIF and attitudes appears inconsistent, with lectures and teaching having less significance than other interpersonal and external factors. Most students felt that it was inadequate and wanted more timetabled contact with substance users. Evidence from Silins et al. [21] supported the provision of structured clinical experience with patients similar to the experiences students were describing. This suggests that such interactive teaching sessions may be successful in ensuring positive PIF and may be well received by students.

Furthermore, interviews revealed that there may be too much reliance on hidden and informal curricula to facilitate PIF. This may mean some students do not receive the same facilitated learning experiences, meaning it may be harder for them to develop positive PIF caring for drug users. Butani et al. [22] found that using patient educators helped students develop their professional values. Therefore, timetabled facilitated learning with drug users may be a way to ensure all students are able to develop positive attitudes.

### 4.4 Implications of the Study and Recommendations

This study has several important implications. It helps medical educators gain insight into medical students' attitudes towards drug users, the factors potentially affecting their PIF towards caring for these patients and how they could better support them to address the identified deficiencies. This aligns with the General Medical Council's Outcomes for Graduates [23, 24] which highlights that graduating doctors are able to manage patients with substance use disorders.

This research suggests that medical school curricula and educators have limited influence on students' knowledge and skills regarding substance use disorders. To ameliorate this, educators should consider students' suggestions for better learning opportunities and prioritise experiential learning on substance use, complemented by structured opportunities for collective reflection. It could help address the stigmatising attitudes held by some students and their reliance on informal and hidden curricula for learning in this area. This research also recommends faculty development initiatives to focus on raising clinical staff awareness of role‐modelling best practices, emphasising the importance of being conscious of their role models in all situations [25] and using ‘reflecting in action’ to make implicit learning explicit [22]. Such measures could positively shape medical students' PIF and help counter the deterioration in attitudes towards drug users as they advance in seniority.

Importantly, these findings extend beyond medical education to all health professional training within Team‐based Care Training (TCT) frameworks. Nurses, pharmacists, social workers, physiotherapists and other allied health professionals encounter substance users regularly and require similar structured education that prioritises experiential learning over theory. TCT programmes should embed shared learning opportunities with service users, facilitate explicit discussion of stigma across disciplines and implement interprofessional faculty development to ensure consistent positive role‐modelling. By addressing these needs across all health professions, we can ensure equitable learning experiences and foster a culture of compassionate, evidence‐based care for this vulnerable population.

## 5 Limitations

The questionnaire had more limited responses than anticipated, probably due to the restrictive nature of the distribution permitted by ethics and the medical schools. It is unknown how many students were aware of the questionnaire; thus, the actual response rate is not possible to determine. However, the sample was broadly representative of medical school cohorts and national medical student demographics [26]. The interviews covered sensitive topics, which may have limited participant openness and depth of insight shared despite the researcher providing a supportive environment.

Additionally, the study was conducted in only two UK medical schools, which may limit generalisability to other healthcare systems internationally. Students with stronger views on substance use may have been more likely to participate, potentially introducing response bias. Finally, this cross‐sectional study captured attitudes at a single time point; longitudinal follow‐up would be valuable to determine whether observed attitudes persist into clinical practice and how they evolve over time.

## 6 Conclusion

While most medical students hold positive attitudes towards individuals who use drugs, concerning stigmatising views emerge during training, particularly declining support for treatment funding and increasing negative perceptions with seniority. Students' PIF is shaped more powerfully by clinical encounters and role models than by formal teaching, highlighting curriculum gaps and overreliance on hidden curricula.

These findings have direct relevance for all health professionals working within interprofessional teams, including nurses, pharmacists, social workers, physiotherapists and other allied health professionals. There is a clear need to formalise what currently depends on chance and ensure equity of learning experiences that equip all health professionals to provide compassionate, evidence‐based care to this vulnerable population.

## Author Contributions

**Holly Melvin:** conceptualization, investigation, writing – original draft, methodology, formal analysis. **Dilmini Karunaratne:** methodology, writing – review and editing, supervision. **Jason W. Boland:** methodology, writing – review and editing, supervision.

## Funding

The authors have nothing to report.

## Ethics Statement

The Hull York Medical School (HYMS) Ethics Committee approval was obtained on 29 November 2021 (REC 21/22 13).

## Conflicts of Interest

The authors declare no conflicts of interest.

## Supporting information


**Appendix S1:** Interview guide.
**Appendix S2:** Qualitative theme descriptions.

## Data Availability

Anonymised data are available from the first author.

## References

[1] Office for Health Improvement & Disparities , “Adult Substance Misuse Treatment Statistics 2023 to 2024: Report. [Internet].GOV.UK. 2024,” [accessed 14 Feb. 25]. Available from, https://www.gov.uk/government/statistics/substance‐misuse‐treatment‐for‐adults‐statistics‐2023‐to‐2024/adult‐substance‐misuse‐treatment‐statistics‐2023‐to‐2024‐report. [Internet].GOV.UK. 2023.

[2] B. James and J. Omoaregba , “Nigerian Medical Students' Opinions About Individuals Who Use and Abuse Psychoactive Substances,” Substance Abuse: Research and Treatment 7 (2013): 121–129. 10.4137/SART.S12129 PMC3682755 23861587

[3] M. Lindberg , C. Vergara , R. Wild‐Wesley , and C. Gruman , “Physicians‐In‐Training Attitudes Toward Caring for and Working With Patients With Alcohol and Drug Abuse Diagnoses,” Southern Medical Journal 99, no. 1 (2006): 28–35. 16466119 10.1097/01.smj.0000197514.83606.95

[4] A. Korszun , S. Dinos , K. Ahmed , and K. Bhui , “Medical Student Attitudes About Mental Illness: Does Medical‐School Education Reduce Stigma?,” Academic Psychiatry 36, no. 3 (2012): 197. 22751821 10.1176/appi.ap.10110159

[5] J. Avery , B. Han , E. Zerbo , et al., “Changes in Psychiatry Residents' Attitudes Towards Individuals With Substance Use Disorders Over the Course of Residency Training,” American Journal on Addictions 26, no. 1 (2016): 75–79. 27749984 10.1111/ajad.12406

[6] I. Wilson , L. S. Cowin , M. Johnson , and H. Young , “Professional Identity in Medical Students: Pedagogical Challenges to Medical Education,” Teaching and Learning in Medicine 25, no. 4 (2013): 369–373. 24112208 10.1080/10401334.2013.827968

[7] S. Sarraf‐Yazdi , Y. Teo , A. How , et al., “A Scoping Review of Professional Identity Formation in Undergraduate Medical Education,” Journal of General Internal Medicine 36, no. 11 (2021): 3511–3521. 34406582 10.1007/s11606-021-07024-9 PMC8606368

[8] F. W. Hafferty , “Professionalism and the Socialization of Medical Students. In: Cruess RL, Cruess SR, Steinert Y, eds. Teaching Medical Professionalism,” in Teaching Medical Professionalism, eds. R. L. Cruess , S. R. Cruess , and Y. Steinert (Cambridge University Press, 2009), 53–73.

[9] B. Burford , “Group Processes in Medical Education: Learning From Social Identity Theory,” Medical Education 46, no. 2 (2012): 143–152. 22239328 10.1111/j.1365-2923.2011.04099.x

[10] J. Carroll , C. Goodair , A. Chaytor , C. Notley , H. Ghodse , and P. Kopelman , “Substance Misuse Teaching in Undergraduate Medical Education,” BMC Medical Education 14, no. 1 (2014): 34. 24533849 10.1186/1472-6920-14-34 PMC3932109

[11] S. Chan Carusone , A. Guta , S. Robinson , et al., ““Maybe if I Stop the Drugs, Then Maybe They'd Care?”—Hospital Care Experiences of People Who Use Drugs,” Harm Reduction Journal [Internet] 16, no. 1 (2019): 16. 30760261 10.1186/s12954-019-0285-7 PMC6373073

[12] L. C. van Boekel , E. P. M. Brouwers , J. van Weeghel , and H. F. L. Garretsen , “Stigma Among Health Professionals Towards Patients With Substance Use Disorders and Its Consequences for Healthcare Delivery: Systematic Review,” Drug and Alcohol Dependence 131, no. 1–2 (2013): 23–35. 23490450 10.1016/j.drugalcdep.2013.02.018

[13] V. John‐Steiner and H. Mahn , “Sociocultural Approaches to Learning and Development: A Vygotskian Framework,” Educational Psychologist 31 (1996): 191–206.

[14] A. Shorten and J. Smith , “Mixed Methods Research: Expanding the Evidence Base,” Evidence‐Based Nursing 20, no. 3 (2017): 74–75. 28615184 10.1136/eb-2017-102699

[15] S. Bunniss and D. Kelly , “Research Paradigms in Medical Education Research,” Medical Education 44, no. 4 (2010): 358–366. 20444071 10.1111/j.1365-2923.2009.03611.x

[16] N. Singleton , “Attitudes to Drug Dependence Results From a Survey of People Living in Private Households in the UK,” Ukdpc.org.uk. (2010) [accessed 14 Feb. 25]. Available from, https://www.ukdpc.org.uk/wp‐content/uploads/Evidence%20review%20‐%20Attitudes%20to%20drug%20dependence_%20survey%20results.pdf.

[17] B. DiCicco‐Bloom and B. Crabtree , “The Qualitative Research Interview,” Medical Education 40, no. 4 (2006): 314–321. 16573666 10.1111/j.1365-2929.2006.02418.x

[18] V. Braun and V. Clarke , “Using Thematic Analysis in Psychology,” Qualitative Research in Psychology 3, no. 2 (2006): 77–101.

[19] T. Stenfors , A. Kajamaa , and D. Bennett , “How to … Assess the Quality of Qualitative Research,” Clinical Teacher 17, no. 6 (2020): 596–599. 32790137 10.1111/tct.13242

[20] G. Papazisis , S. Siafis , I. Tsakiridis , I. Koulas , T. Dagklis , and D. Kouvelas , “Prevalence of Cannabis Use Among Medical Students: A Systematic Review and Meta‐Analysis,” Substance Abuse: Research and Treatment 12 (2018): 117822181880597. 10.1177/1178221818805977 PMC6194916 30349282

[21] E. Silins , K. Conigrave , C. Rakvin , T. Dobbins , and K. Curry , “The Influence of Structured Education and Clinical Experience on the Attitudes of Medical Students Towards Substance Misusers,” Drug and Alcohol Review 26, no. 2 (2007): 191–200. 17364855 10.1080/09595230601184661

[22] L. Butani , C. Sweeney , and J. Plant , “Effect of a Patient‐Led Educational Session on Pre‐Clerkship Students' Learning of Professional Values and on Their Professional Development,” Medical Education Online 25, no. 1 (2020): 1801174. 32730189 10.1080/10872981.2020.1801174 PMC7482741

[23] General Medical Council , “Outcomes for Graduates,” Gmc‐uk.org. (2020) [accessed 14 Feb. 25]. Available from, https://www.gmc‐uk.org/education/standards‐guidance‐and‐curricula/standards‐and‐outcomes/outcomes‐for‐graduates/outcomes‐for‐graduates/structure‐and‐overarching‐outcome#overarching‐outcome‐for‐graduates.

[24] Gmc‐uk.org , (2024), [accessed 14 Feb. 25]. Available from, https://www.gmc‐uk.org/professional‐standards/the‐professional‐standards/good‐medical‐practice/the‐duties‐of‐medical‐professionals‐registered‐with‐the‐gmc.

[25] S. Cruess , R. Cruess , and Y. Steinert , “Role Modelling—Making the Most of a Powerful Teaching Strategy,” BMJ 336, no. 7646 (7646): 718–721. 10.1136/bmj.39503.757847.BE PMC2276302 18369229

[26] Medical Schools Council , “Selection Alliance 2023 Report‐ An update on the Medical Schools Council's work in selection and widening participation,” (2023). [accessed 4 Jun. 25]. Available from, https://www.medschools.ac.uk/media/3125/selection‐alliance‐update‐2023.pdf.

