# Supplementary material for: ‘We All Try to Imagine What It's Like for Someone to Be Dependent’: Attitudes Towards Drug Users Among Medical Students in the United Kingdom and Its Impact on Their Professional Identity Formation
Source: Clin Teach. 2026 Jul 7;23(4):e70473. doi: 10.1111/tct.70473 (PMC13339741; doi:10.1111/tct.70473)
Supplement: Supplementary file 1 — Appendix S1: Interview guide. Appendix S2: Qualitative theme descriptions. [file TCT-23-e70473-s001.docx]

**Supplementary Material:**

(The Clinical Teacher Early Career Scholarship Submission)

*Title:*

*“We all try to imagine what it’s like for someone to be dependent” : A*ttitudes towards drug users among medical students in the United Kingdom and its impact on their professional identity formation

**Appendix 1:**

**Interview Guide**

Introduction

This interview aims to explore your opinions on illicit drug use. We will be discussing drug dependence, your experiences with patients who use illicit drugs, your experiences of knowing people who use illicit drugs, and your ideas on current teaching of illicit drug use. Please remember that you do not have to answer any questions if you do not feel comfortable. I just want to remind you that we are obliged to inform the support services at your medical school if you disclose personal illicit drug use. Also to remind you that this interview will be recorded.

**Demographic questions:**

What Medical school do you attend?

What year are you in at Medical school?

What is your age?

How would you describe your gender?

Did you complete a recognised widening participation/widening access scheme prior to entering medical school?

Did you receive free school meals at school?

**Main questions:**

1. What is your understanding of illicit drug dependence?
2. What do you think about patients who use illicit drugs?
3. What have your experiences with patients who use illicit drugs been like?
4. How have these experiences influenced how you view patients who use illicit drugs?
5. Other influences
6. What do you think about the idea that patients who use illicit drugs are less deserving of treatment and care?
7. How do you think doctors influenced your opinions patients who use illicit drugs? Could you explain this more?
8. How do you think other health care professionals influenced your opinions patients who use illicit drugs? Could you explain this more?
9. Have you had any experience of illicit drug use affecting someone you know? Has this affected how you feel about patients who use illicit drugs?
10. How do you feel about current teaching of illicit drug use at medical school? Why is that?
11. What do you think could be done differently?
12. How do you think a good doctor should look after patients who use illicit drugs?

**Appendix 2:**

Qualitative theme descriptions

| Theme | Description |
| --- | --- |
| **Theme 1: Factors shaping perceptions and attitudes towards patients who use or are dependent on illicit drugs** | A diversity of personal, interpersonal, educational and contextual factors determine how individuals who use drugs are perceived by medical undergraduates. |
| **Theme 2: Students’ understanding of the nature of addiction and illicit drug use** | Students’ understanding of addiction and illicit drug use centres around social circumstances, relating illicit drug dependence to other health conditions and societal stigma. |
| **Theme 3: Perceived gaps and opportunities in medical school teaching on illicit drug use** | There is a wide range of ideas on the quality and quantity of university teaching on drug use and dependence, including changes students would like. |
| **Theme 4: Student aspirations for caring for people who use illicit drugs** | Student aspirations underscored their commitment to developing into practitioners who provide compassionate and inclusive care for individuals using illicit drugs. |
